# Comprehensive study on the Python-based regression machine learning models for prediction of uniaxial compressive strength using multiple parameters in Charnockite rocks

**DOI:** 10.1038/s41598-024-58001-1

**Published:** 2024-03-28

**Authors:** Sowmya Kochukrishnan, Premalatha Krishnamurthy, Yuvarajan D., Nandagopal Kaliappan

**Affiliations:** 1 Department of Civil Engineering, Anna University, Chennai, Tamil Nadu India; 2 Department of Mechanical Engineering, Saveetha School of Engineering, SIMATS, Saveetha University, Chennai, Tamil Nadu India; 3 Department of Mechanical Engineering, Haramaya Institute of Technology, Haramaya University, Dire Dawa, Ethiopia

**Keywords:** Python model, Machine learning techniques, Regression, Uniaxial compressive strength, Indirect parameters, Engineering, Civil engineering

## Abstract

The strength of rock under uniaxial compression, commonly known as Uniaxial Compressive Strength (UCS), plays a crucial role in various geomechanical applications such as designing foundations, mining projects, slopes in rocks, tunnel construction, and rock characterization. However, sampling and preparation can become challenging in some rocks, making it difficult to determine the UCS of the rocks directly. Therefore, indirect approaches are widely used for estimating UCS. This study presents two Machine Learning Models, Simple Linear Regression and Step-wise Regression, implemented in Python to calculate the UCS of Charnockite rocks. The models consider Ultrasonic Pulse Velocity (UPV), Schmidt Hammer Rebound Number (N), Brazilian Tensile Strength (BTS), and Point Load Index (PLI) as factors for forecasting the UCS of Charnockite samples. Three regression metrics, including Coefficient of Regression (R^2^), Root Mean Square Error (RMSE), and Mean Absolute Error (MAE), were used to evaluate and compare the performance of the models. The results indicate a high predictive capability of both models. Notably, the Step-wise model achieved a testing R^2^ of 0.99 and a training R^2^ of 0.988 for predicting Charnockite strength, making it the most accurate model. The analysis of the influential factors indicates that UPV plays a significant role in predicting the UCS of Charnockite.

## Introduction

Rocks and their structures require careful planning to prevent loss of life and economic damage from human error. In civil engineering, mining, cave mining, tunneling applications, and other related fields, evaluating rock quality heavily relies on Uniaxial Compressive Strength (UCS), a crucial parameter. The primary goal of conducting the UCS test is to determine the strength and properties of the rock. UCS testing can be laborious and costly, necessitating the preparation of meticulously crafted samples, particularly in the case of soft and jointed rock formations. As a result, researchers have proposed empirical equations that relate UCS to other parameters. Past studies have investigated correlations between UCS and other rock properties, and various researchers have proposed empirical equations for metamorphic rocks, which are listed in Table 1. Since the chosen rock type for the present study is a metamorphic rock, only empirical equations for metamorphic rocks are considered.

**Table 1:** Equations for estimation of UCS using PLI, BTS, N

Source	Equation	Reference	Rock type
Basu and Kamran (2010)	UCS = 11.103Is(50) + 37.659	P1	Metamorphic
Kahraman and Gunaydin (2009)	UCS = 18.45Is(50) + 13.63	P2	Metamorphic
Singh and Singh (1993)	UCS = 23.37Is(50)	P3	Metamorphic
Singh et al. (2012)	UCS = 22.8Is(50)	P4	Metamorphic
Diamantis et al. (2009)	UCS = 19.79Is(50)	P5	Metamorphic
Tandon and Gupta (2015)	UCS = 4.792Is(50) + 44.37	P6	Metamorphic
Diamantis et al. (2009)	UCS = 17.81Is(50)^1.06^	P7	Metamorphic
Diamantis et al. (2009)	UCS = 21.54Is(50) − 6.02	P8	Metamorphic
Fereidooni (2016)	UCS = 24.36Is(50) − 2.14	P9	Metamorphic
Fereidooni (2016)	UCS = 10.03BTS + 55.19	B1	Metamorphic
Yilmaz and Sendir (2002)	UCS = exp(0.818 + 0.059N)	S6	Metamorphic
Gupta (2009)	UCS = 0.64N + 37.5	S7	Metamorphic
Fereidooni (2016)	UCS = 0.02N^2.28^	S8	Metamorphic
Torabi et al. (2010)	UCS = 0.0465N^2^ − 0.1756N + 27.682	S9	Metamorphic

Several researchers have proposed empirical equations for predicting different rock types’ unconfined compressive strength (UCS) using physio-mechanical parameters and non-destructive tests. For instance, Fakir et al.^1^ developed equations for the granitoid rocks of South Africa, while Habib et al.^2^ established excellent correlations for sedimentary rocks in Algeria. Jalali et al.^3^ prepared linear regression equations for igneous and metamorphic rocks from different locations in Iran using block punch index, cylindrical punch index, and UCS tests. Similarly, Kurtulus et al.^4^, Son and Kim^5^, Aldeeky and Hattamleh^6^, Arman and Paramban^7^, Bolla and Paronuzzi^8^, and Chawre^9^ found promising results in relating UCS to NDT tests such as Schmidt hammer, Ultrasonic pulse velocity (UPV), and total sound-signal energy for different rock types. Furthermore, Mishra et al.^10^ conducted mechanical, physical, and petrological studies on rock types in India and classified them accordingly. Aladejare et al.^11^ compiled a dataset of experimental correlations between uniaxial compressive strength (UCS) and various other rock properties based on published literature, which can help in selecting a suitable regression equation for estimating the UCS of a rock site.

Various studies have established predictive models for determining the UCS of rocks using soft computing techniques. Wang et al.^12^ developed a model using a random forest algorithm that showed consistent results with laboratory tests. Dadhich et al.^13^ analyzed the efficacy of an ML model based on various features and concluded that random forest regression was the optimal method. Wang et al.^14^ applied two machine learning models using non-destructive and petrographic studies and showed that the extreme gradient boosting model outperformed the random forest model in predicting UCS. Tang et al.^15^ established a predictive model using an improved least squares tree algorithm that demonstrated the model’s usefulness in engineering applications. Fattahi^16^ introduced a new relevance vector regression (RVR) method enhanced by two algorithms to forecast the UCS of weak rocks. They found that the RVR optimized by the harmony search algorithm outperformed the one optimized by the cuckoo search. Lei et al.^17^ conducted a comparative study of six prediction models that were hybrid and based on the BP neural network, along with six optimization algorithms using swarm intelligence. They proved that FA-BP was the best model among others in predicting UCS.

Several studies have demonstrated the effectiveness of Artificial Neural Networks (ANN) and Machine Learning (ML) in predicting the UCS. Momeni et al.^18^ showed that particle swarm optimization-based ANN predictive model outperformed conventional ANN techniques in predicting UCS through direct and indirect estimation. Abdelhedi et al.^19^ demonstrated that the combination of multiple linear regression and ANN effectively indicates the UCS values of carbonate rocks and mortar by correlating porosity, density, and ultrasonic measurements with UCS. Ozdemir^20^ utilized artificial intelligence-based age-layered population structure genetic programming (ALPS-GP) and an artificial neural network (ANN) to estimate the unconfined compressive strength (UCS) and found both methods to be influential. Azarafza et al.^21^ proposed a DNN model and demonstrated its efficacy in obtaining the strength of marlstone. The model was also verified using classifiers like support vector machine, logistic regression, decision tree, loss function, MAE, MSE, RSME, R-square, etc. Wei et al.^22^ used the artificial neural network (ANN) approach to estimate the unconfined compressive strength (UCS) of sedimentary rocks at the Thar Coalfields. Their findings indicated that the Brazilian tensile strength had the most significant influence on UCS estimation. Fang et al.^23^ predicted equations based on various training algorithms and established the supremacy of the ANFIS model over the other models considered. Gupta and Nagarajan^24^, Hassan and Arman^25^, Liu et al.^26^, Shahani et al.^27^, and Qiu et al.^28^ studied the performance of different machine learning models in predicting UCS. They suggested the best model based on factors such as absolute error, root mean square error, coefficient of determination, etc.

Over time, multiple techniques have been developed to predict the rocks’ strength accurately. These techniques have proven effective for different types of rocks. However, this study aims to introduce two simple machine-learning methods, the linear regression model and the step-wise regression model, implemented in Python to estimate the strength of Charnockite rocks. The models analyze the parameters BTS, PLI, N, and UPV to predict the strength of Charnockite rocks. The models also provide a matching procedure to assess the strength of rocks in Chennai City, Tamil Nadu, India.

## Materials and methods

### Location of the study site

**Figure 1:**
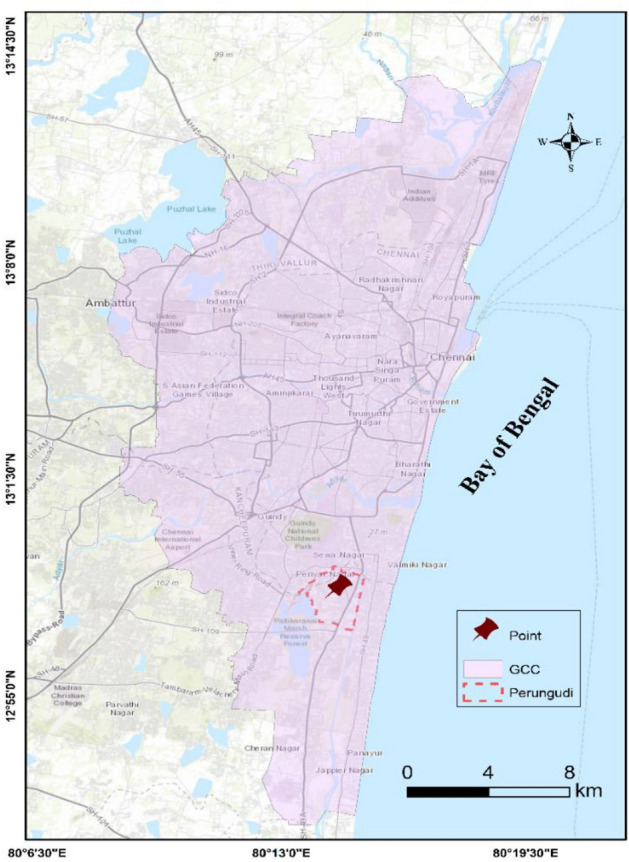
Map showing the location of Perungudi in Chennai.

The Perungudi region, situated in the southern part of Chennai, is a bustling commercial hub with numerous construction projects. The area is home to Charnockite and Granite rocks, widely found throughout Chennai. This article presents an alternative method for estimating the UCS of Charnockite rocks in the Perungudi region, which can also be applied to other parts of the city. Figure 1 displays the location of the Perungudi region.

### Rock material testing and database

#### Petrographic analysis

**Figure 2:**
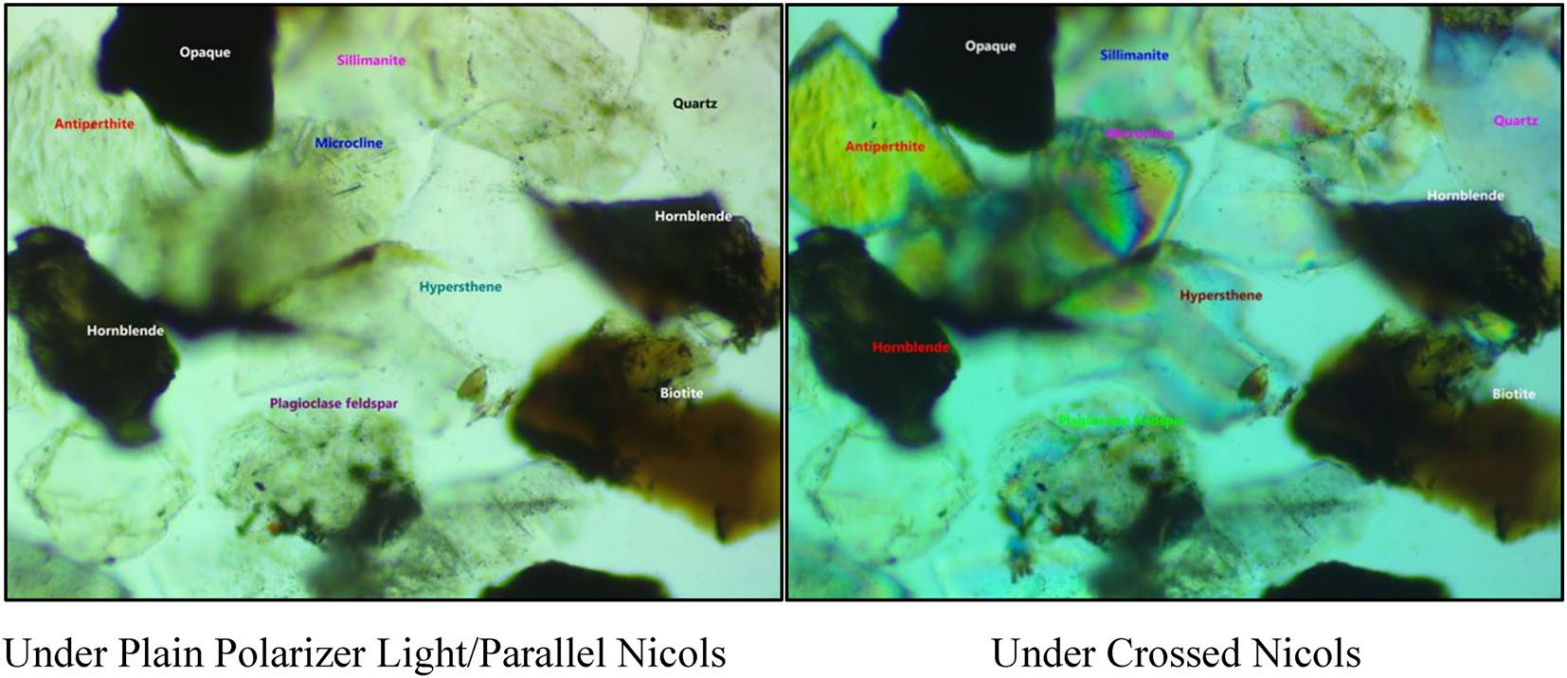
Images of rock under a petrographic microscope.

Macroscopic and microscopic observations were conducted to determine the type of rock. The rocks appeared dark grey to black in their fresh state, with a fine-to-medium-grained texture and an equigranular granoblastic homogeneous fabric without layering. It was difficult to distinguish the dark grey plagioclase from the mafic portion of the rock, and the presence of hornblende gave it a black hue. Thin sections of basic granulites exhibited granulitic texture, with minerals interfering with each other’s growth. The common minerals constituting the basic granulites were labradorite, hypersthene, and augite, with a constant association of secondary hornblende, sometimes prepondering over the pyroxenes. Black opaque was present in negligible amounts, along with biotite and apatite. The development of faint gneissose structure due to the sublinear arrangement of mafic constituents was observed in some of the slides. The dark color of Charnockite is caused by thin greenish or yellowish-brown veins and stringers throughout the rock, particularly in the feldspars but also in quartz and other minerals. Images of rocks under a petrographic microscope are displayed in Fig. 2. The primary and minor minerals observed from thin section analysis are shown in Table 2.

**Table 2:** Findings from the analysis of rocks under a thin section.

Mineral	Percentage
Plagioclase feldspar	32
Quartz	35
Hypersthene and Hornblende	30
Stained Quartz	< 0.5
Mica—Biotite	< 0.5
Mica—Muscovite	< 0.2
Secondary Mineral—Sillimenite	< 0.5
Rock type—Charnockite	

### Laboratory testing methods

**Figure 3:**
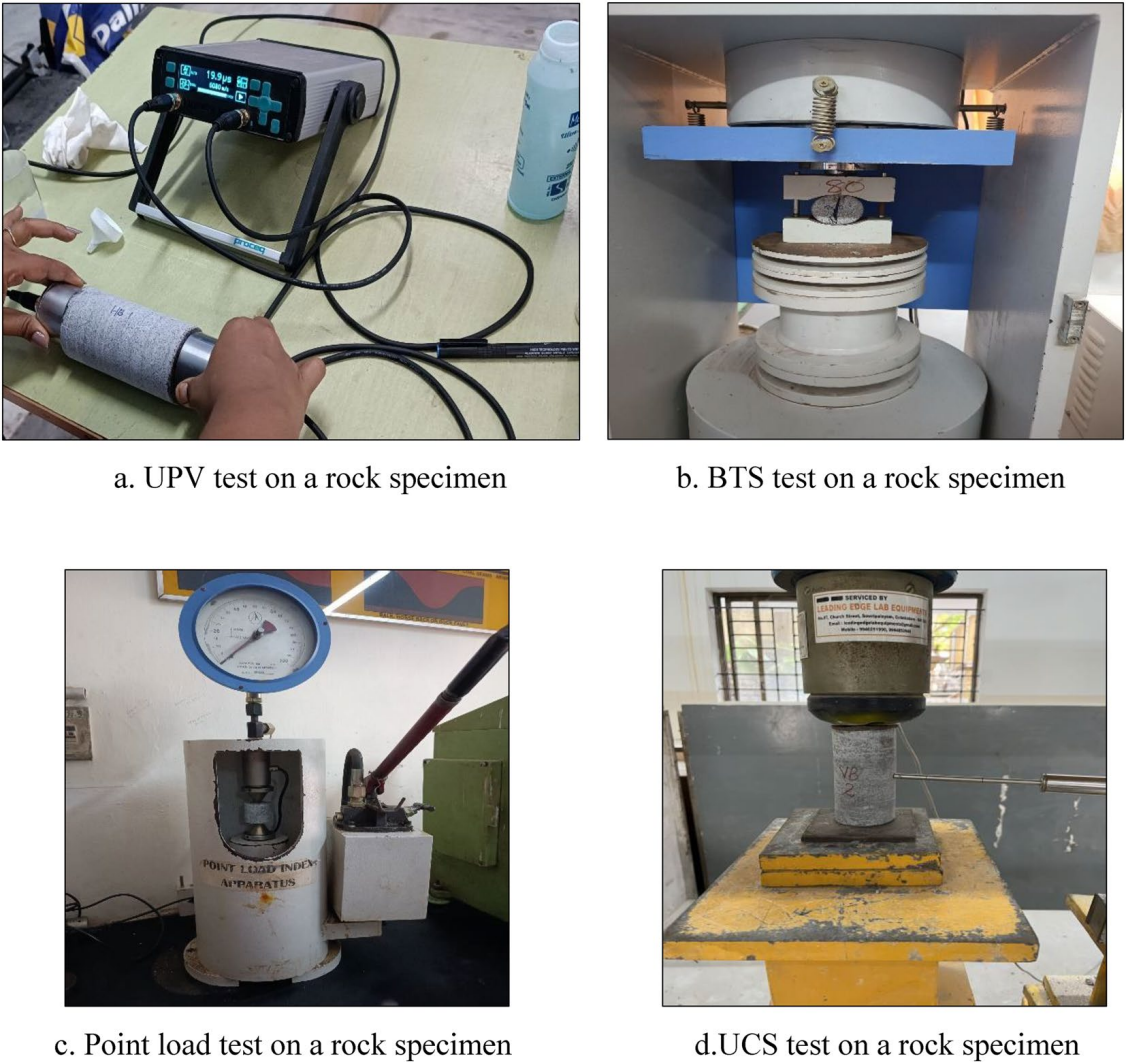
Laboratory tests on a rock specimen.

**Figure 4:**
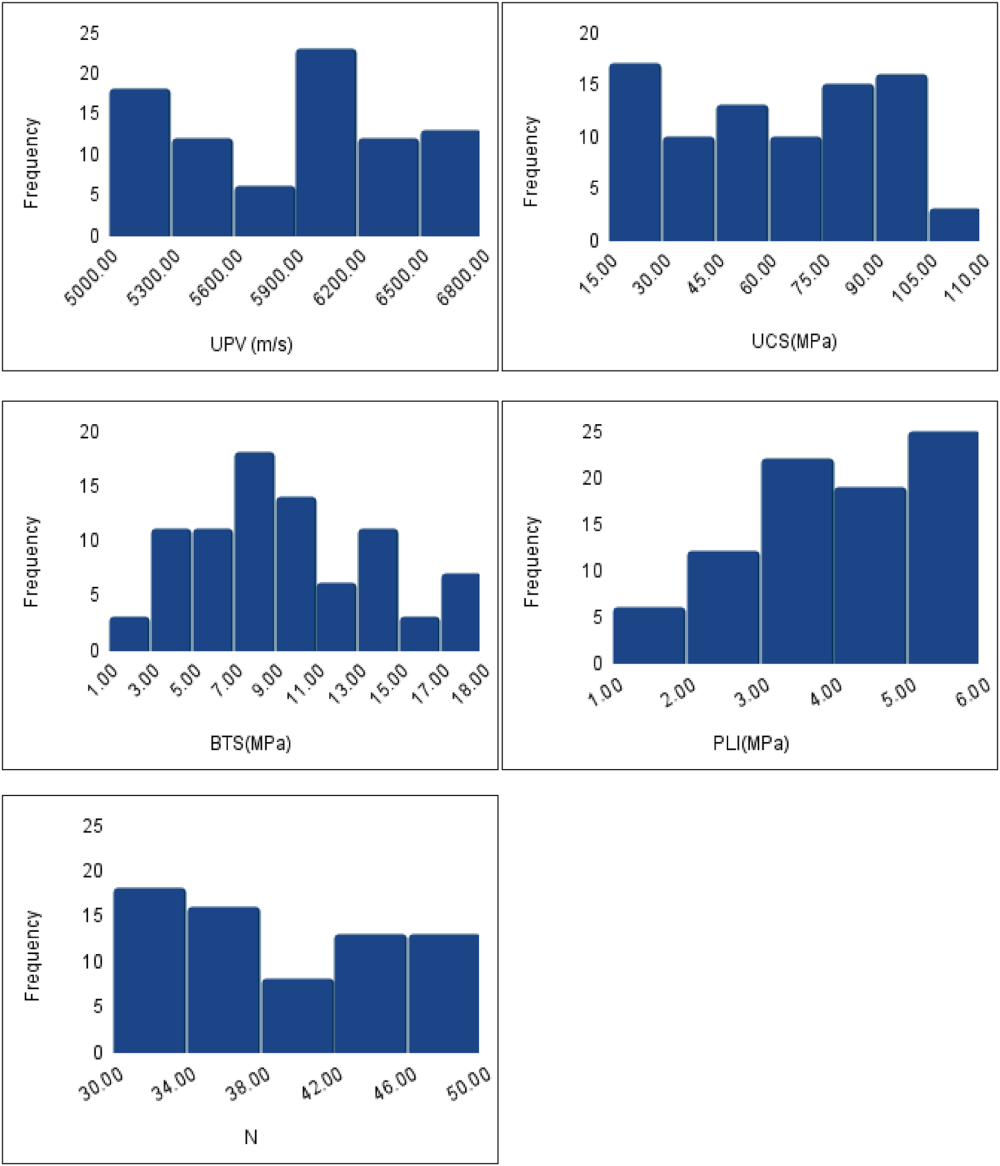
Plot of the properties of Charnockite rocks.

Various properties and parameters of rocks, including index properties, physical parameters, and destructive and non-destructive parameters, have been found to correlate with the UCS of different types of rocks. Equations have been established with high levels of accuracy by Bagherpour et al.^29^ and Daoud et al.^30^. However, Aladejare^31^ suggests using these equations only for the same rock types they were developed for. To investigate the UCS of rocks in the Perungudi region in Chennai, 84 specimens were collected from different locations. These specimens were then transported to the laboratory for various tests, including the UCS test, BTS test, PLI test, SHN, and UPV, following ASTM standards. The specimens were prepared and tested by ASTM D4543-19^32^ and ASTM D7012^33^ standards. Procedures for determining pulse velocities, rock hardness by rebound hammer method, Brazilian tensile strength, and point load index were suggested by ASTM D2845-08^34^, ASTM D5873-95^35^, ASTM D3967-95a^36^, and ASTM D5731-02^37^, respectively. All tests reported in this paper adhered to the standards (Fig. 3). The results of the various tests performed in the laboratory are shown in Table 3. The table provides statistical information on the properties of the rocks, which was used as a database for the study. Figure 4 depicts a histogram plot showing the variation of the properties.

**Table 3:** Engineering properties of Charnockite rocks.

Property	Range	Mean	Standard deviation
PLI (MPa)	1.8–5.98	4.05	1.248
BTS (MPa)	2.25–17.88	9.49	4.17
Rebound number	24–50	37.76	7.44
UPV (m/s)	5008–6721	5858.49	525.78
UCS (MPa)	16.16–109.16	62.17	28.03

### Regression ML models using Python

In a recent study, Liu et al.^26^ examined the UCS prediction capabilities of three different boosting machine models: adaptive boosting, category gradient boosting, and extreme gradient boosting. They compared the models’ performance using five regression metrics. Another study by Xu et al.^38^ introduced a novel prediction model called the SSA-XGBoost model. This model was more effective in predicting the UCS of rocks than six other models evaluated using RMSE, correlation coefficient, MAE, and variance interpretation. The paper presents two machine learning-based regression models, namely, Linear Regression and Step-wise Regression, that can predict the UCS of Charnockite rocks. The models are constructed using 84 sample data from 4 different parameters (UPV, N, BTS, and PLI) to find the UCS value of the Charnockite rock samples. In both models, 80% of the data are trained with the help of Python supported machine learning techniques like (scipy, numpy, pandas, seaborn, and matplotlib) and with those trained data, the model is fitted in such a way to predict the coefficient and intercept of the data. These findings are necessary to form the linear regression equation, and the r value is obtained from that. The performance of the models was evaluated, and a superior UCS estimation model was reported.

### Linear regression ML model

Linear regression is the most widely used machine learning model. The Scikit-Learn module is used in Python to build, train, and test the linear regression model. To estimate the unconfined compressive strength (UCS) of Charnockite rocks, we utilize the following rock properties: UPV, N, BTS, and PLI. These properties are extracted from a dataset of 84 Charnockite rock samples and fed into the Jupyter Notebook for prediction. To understand the dataset better, a pair plot can be generated. The dataset has been split into two distinct sets: one for training purposes and another for testing. This study used a split of 80:20, with 80% of the dataset allocated for training and the remaining 20% reserved for testing. A linear regression machine learning model was then created and trained using the segregated data. To simplify the process, sci-kit-learn was utilized. Afterward, the machine learning model was applied to the test data set to generate predictions. Scatter plots were created to compare the predicted values with the actual values. To visually evaluate the performance of the model, the residuals were plotted. Python code has been developed considering all four parameters. All 4 data have been considered for both test and train datasets. After training the model with the train data, the test data of variables (UPV, N, BTS, and PLI) are introduced to the trained model to determine the predicted UCS. Then, the model calculates the correlation coefficient by comparing the predicted UCS and the test value of the observed UCS. To represent the findings visually, Python's graphical options present them in chart and plot formats.

#### Python code for linear regression model

Import matplotlib.pyplot as plt

import pandas as pd

import numpy as np

import scipy as sp

import seaborn as sns

plt. style.use('plot)

raw_data = pd.read_csv('R2model.csv')

x = raw_data[['UPV']]

y = raw_data['UCS']

from sklearn.model_selection import train_test_split

x_train, x_test, y_train, y_test = train_test_split(x, y, test_size = 0.9)

from sklearn.linear_model import LinearRegression

model = LinearRegression()

model.fit(x_train, y_train)

print(model.coef_)

print(model.intercept_)

line = f'UCS = {model.coef_}UPV + ({model.intercept_})'

print(line)

#UPV = x_train

#UCS = y_train

#fig, ax = plt.subplots()

#ax.plot(UPV, UCS, linewidth = 0, marker = 's', label = 'Training Data points')

#ax.plot(UPV, model.intercept_ + model.coef_ * UPV, label = line)

#ax.set_xlabel('UPV')

#ax.set_ylabel('UCS')

#ax.legend(facecolor = 'white')

#plt.show()

m = pd.DataFrame(model.coef_, x.columns, columns = ['Coeff'])

print(m)

predictions = model.predict(x_test)

r = sp.stats.linregress(y_test, predictions)

print(r)

slope, intercept, r, p, stderr = sp.stats.linregress(y_test, predictions)

line = f'r = {r:.2f}'

fig, ax = plt.subplots()

ax.plot(y_test, predictions, linewidth = 0, marker = 's', label = 'Test Data points(UPV)')

ax.plot(y_test, intercept + slope * y_test, label = line)

ax.set_xlabel('Observed UCS')

ax.set_ylabel('Predicted UCS')

ax.legend(facecolor = 'white')

plt.show()

#plt.scatter(y_test, predictions)

#plt.show()

plt.hist(y_test - predictions)

plt.show()

from sklearn import metrics

a = metrics.mean_absolute_error(y_test, predictions)

line = f'MAE = {a}'

print(line)

b = metrics.mean_squared_error(y_test, predictions)

line = f'MSE = {b}'

print(line)

c = np.sqrt(metrics.mean_squared_error(y_test, predictions))

line = f'SQRT(MSE) = {c}'

print(line)

### Step-wise regression ML model

Step-wise regression is a method that involves step-by-step inclusion or exclusion of variables to create a regression model that precisely explains the data with the minimum number of essential variables. This approach automatically selects the most significant variables and excludes the insignificant ones, making it superior to many other regression techniques. Initially, all four variables are considered, and at each step, the most insignificant variable is eliminated to provide a better result and to determine the significance of the data.

The data array must be defined and converted to a data frame using NumPy and pandas packages to perform step-wise regression. The Sequential Feature Selector function from the mixed package is used to conduct step-wise regression, and the chosen features are specified using the k_features parameter. Once the step-wise regression is complete, the desired features are used to examine the characteristics of the data. A data frame consisting solely of the selected components and the k_feature_names_ property is then created. The dataset is divided into training and testing sets using the train_test_split method provided by the scikit-learn library. Then, a logistic regression model is fitted based on the chosen features. Finally, the accuracy_score function from the sklearn library is used to assess the model’s performance. The sklearn module of Python will handle the computational complexity, so even in real-time predictions or large-scale application usage, it will be feasible and effective since this model is used for fitting regression models with predictive models. It is carried out automatically. Each step adds or subtracts the variable from the set of explanatory variables. The approaches for stepwise regression are forward selection, backward elimination, and bidirectional elimination.

In this method, Ultrasonic pulse velocity (UPV) and Schmidt Hammer Rebound Number (N) have been eliminated in each step, and the model predicted Brazilian Tensile Strength (BTS) and Point Load Index (PLI) are the significant variables to determine the result.

#### Python code for step-wise regression model

Import numpy as np

import statsmodels.api as sm

import pandas as pd

import scipy as sp

import matplotlib.pyplot as plt

plt.style.use('ggplot')

from sklearn.linear_model import LinearRegression

data = pd.read_csv('R2model.csv')

x_columns = data[['BTS,' 'PLI']]

y = data['UCS']

def get_stats():

results = sm.OLS(y, x_columns).fit()

print(results.summary())

get_stats()

linear_model = LinearRegression()

linear_model.fit(x_columns, y)

from sklearn.model_selection import train_test_split

x_train, x_test, y_train, y_test = train_test_split(x_columns, y, test_size = 0.2)

y_pred = linear_model.predict(x_test)

print("Prediction for UCS is ", y_pred)

from sklearn import metrics

a = metrics.mean_absolute_error(y_test, y_pred)

line = f'MAE = {a}'

print(line)

b = metrics.mean_squared_error(y_test, y_pred)

line = f'MSE = {b}'

print(line)

c = np.sqrt(metrics.mean_squared_error(y_test, y_pred))

line = f'SQRT(MSE) = {c}'

print(line)

r = sp.stats.linregress(y_test, y_pred)

print(r)

slope, intercept, r, p, stderr = sp.stats.linregress(y_test, y_pred)

line = f'r = {r:.2f}'

fig, ax = plt.subplots()

ax.plot(y_test, y_pred, linewidth = 0, marker = 's', label = 'Test Data points')

ax.plot(y_test, intercept + slope * y_test, label = line)

ax.set_xlabel('Observed UCS')

ax.set_ylabel('Predicted UCS')

ax.legend(facecolor = 'white')

plt.show()

## Results and discussion

Two machine learning models, Linear Regression and Step-wise Regression, were developed using the majority (80%) of the dataset for training, and the remaining 20% was utilized for testing the model. The model will initially train the data of all four variables to predict the linear regression equation using the Python functionalities (scipy, numpy, pandas) to find the r value. After that, the sklearn functionality will handle the multicollinearity issue with the trained model and predict the UCS with the help of test data.

Regression metrics, including R2, MAE, and RMSE, were used to evaluate the accuracy of the models. The database was provided by sampling and testing the rocks obtained from the Perungudi region in Chennai. The following datasets were used for training and testing to validate the models. These data are representative of the Charnockite population, as seen in the various literature datasets. UPV range is 5016–6721 m/s, UCS range is 16.72–109.16 MPa, SHN range is 24–50, PLI range is 1.81–5.98 MPa and BTS range is 2.25–17.88 MPa.

**Figure 5:**
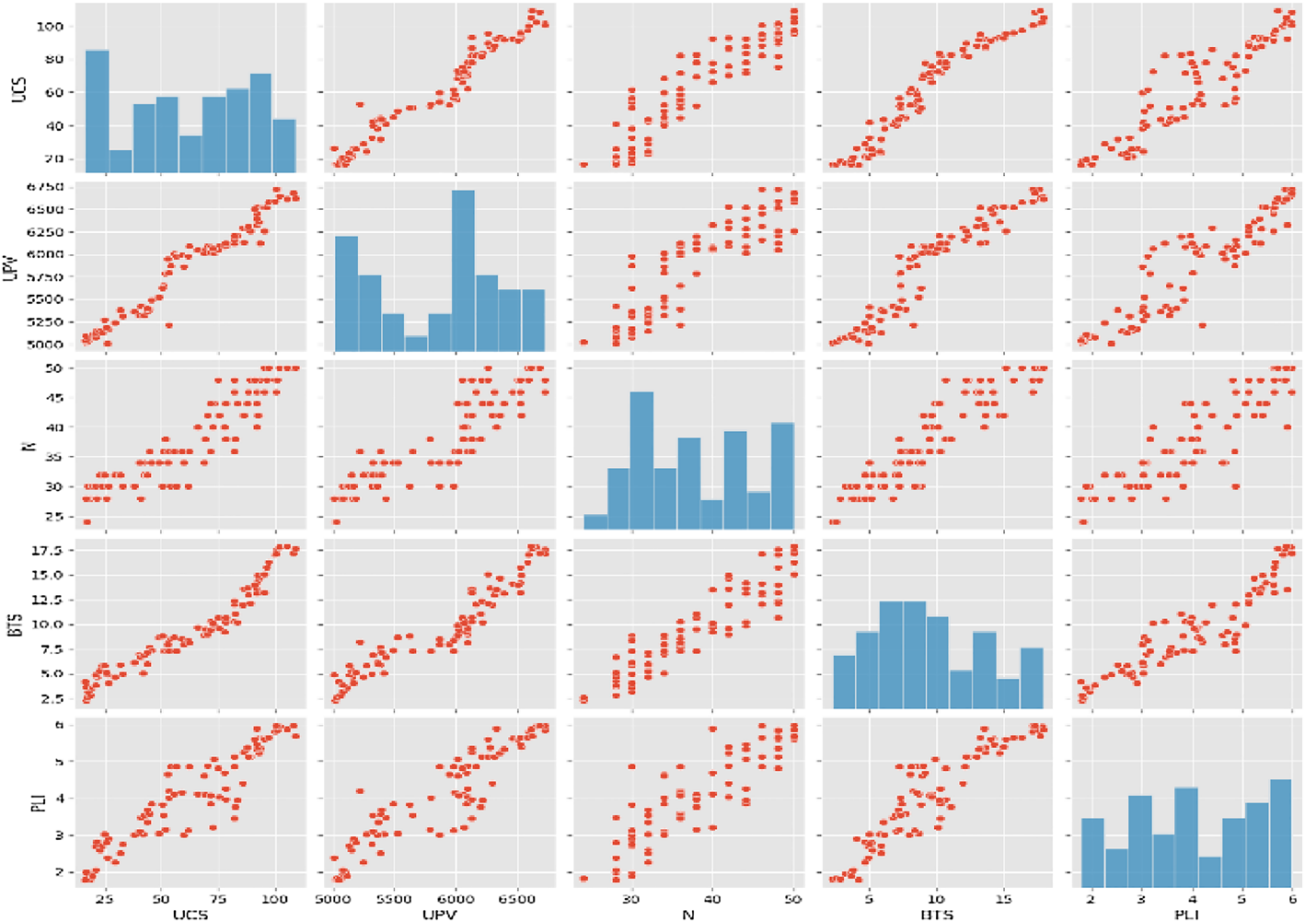
Pair plot of the database.

**Figure 6:**
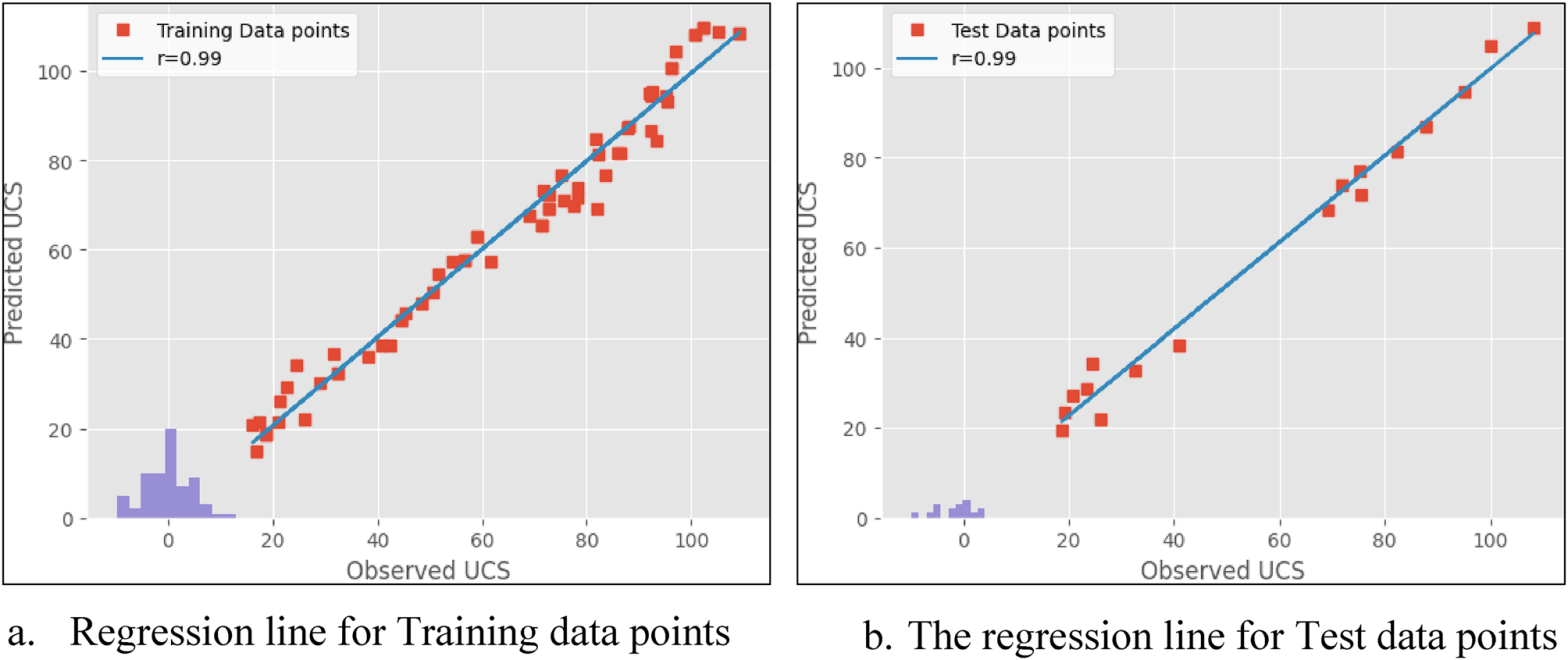
Regression line for ML linear regression model.

**Figure 7:**
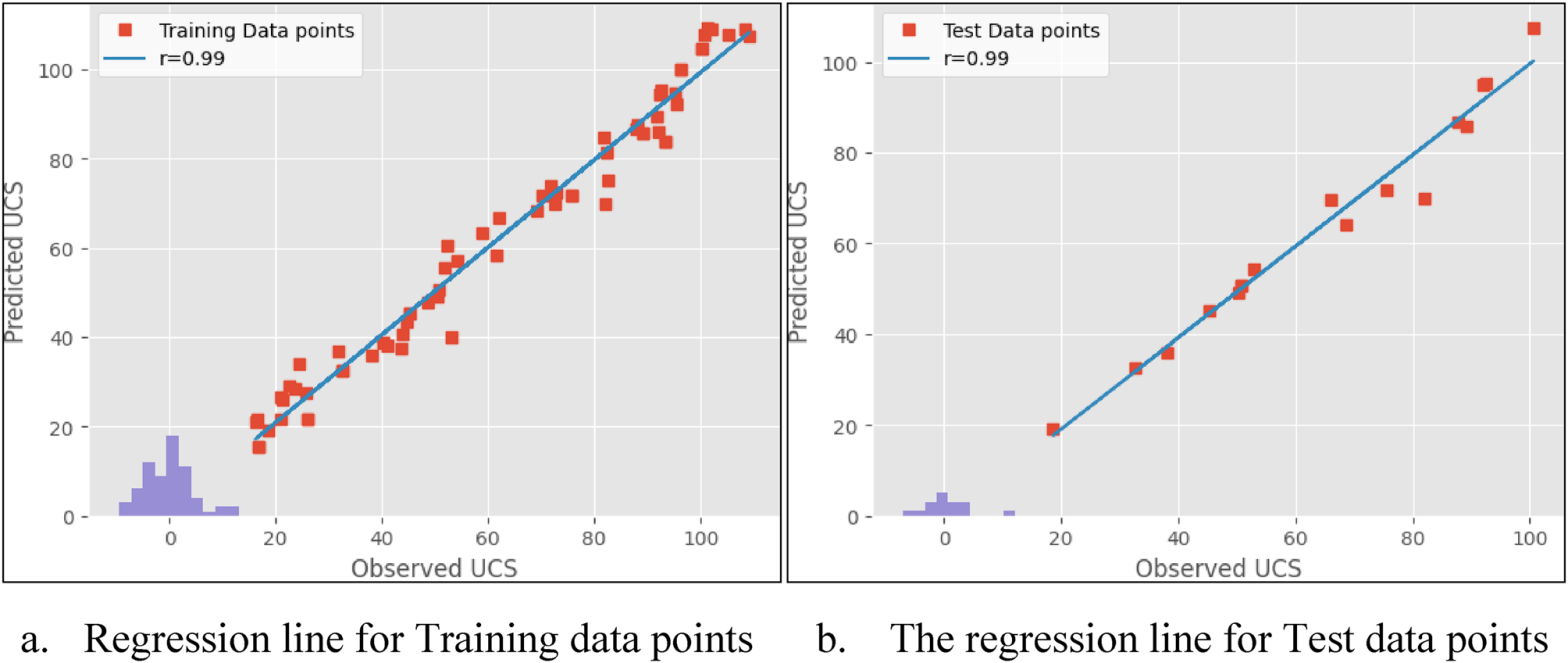
Regression line for step-wise regression.

The results obtained from the experiments are presented in Figs. 5, 6 and 7. Both models proved to be highly accurate methods for predicting the UCS of the rock. Figure 5 shows the scatter distributions between the variables in the linear regression model. Figure 6 shows the predicted values of the UCS against the observed values during the training and testing of the linear regression model. Figure 7 shows the predicted values of the UCS against the experimental values during training and testing of the step-wise regression model. The experiments were carried out by the ASTM standards on 84 Charnockite samples that were recovered from various parts of the Perungudi region. The samples were tested in the laboratory to assess properties such as UCS, UPV, N, BTS, and PLI. The results from the experiments were used to evaluate the performance of the models. The predictive values that the linear and step-wise regression models prepared were compared with the observed values by regression. The results showed that both models correlated well with the observed values.

Table 4 provides the empirical equations for estimating UCS with individual parameters. The analysis showed a good correlation between all parameters and the UCS, with UPV having the highest correlation and N having the lowest. The estimated R^2^ for the linear regression model was 0.98 and 0.986 for training and test datasets, indicating high accuracy of data overlap. Similarly, for the step-wise regression model, the estimated R^2^ was 0.988 and 0.99 for training and test datasets, indicating high accuracy. The straight line in both scatter plots suggests that the machine-learning models have accurately predicted the UCS values. The residuals from the model’s histogram show that they are typically distributed, demonstrating its efficiency.

**Table 4:** Equation for UCS estimation from various parameters.

Parameter involved	Linear regression	Correlation coefficient (R^2^)
UPV	UCS = 0.052*UPV − 244.46	0.95
BTS	UCS = 6.54*BTS − 1.58	0.92
N	UCS = 3.14*N − 54.03	0.84
PLI	UCS = 21.06*PLI − 22.29	0.85

Table 5 presents the regression metrics of the linear regression model and step-wise regression model. The indices show the variations between the values that were predicted and the ones that were observed. According to the analysis, for the training dataset in the linear regression model, the estimated values for MAE and RMSE were 3.41 and 4.41, respectively. For the test dataset, the values were 2.90 and 3.83, respectively. Similarly, for the step-wise regression model, the estimated MAE and RMSE for the training dataset were 3.63 and 4.66, respectively, and for the test dataset, the values were 2.71 and 3.60, respectively. The MAE and RMSE values were lower in the step-wise regression model than in the linear regression model. The reduction in the MAE and RMSE values indicated the high accuracy of the prediction capacity of the step-wise regression model.

**Table 5:** Regression metrics table for the Models.

Model	Training data			Test data		
	R^2^	MAE	RMSE	R^2^	MAE	RMSE
Linear regression	0.98	3.41	4.41	0.986	2.90	3.83
Step-wise regression	0.988	3.63	4.66	0.99	2.71	3.60

## Conclusions

The strength of rock, along with other properties, plays a crucial role in civil projects. Many properties can be conveniently tested in both the laboratory and the field. In this research, we performed tests to measure the rock properties in the laboratory. We determined properties such as UPV, N, BTS, and PLI for Charnockite samples obtained from the Perungudi region in Chennai. We used Python to implement the Linear Regression ML model and Step-wise Regression to predict the Uniaxial Compressive Strength (UCS). Petrographic studies confirmed the rock as Charnockite rocks, displaying high percentages of Quartz, Feldspar, Hypersthene, and Hornblende, with slight traces of mica and Sillimanite. The statistical analysis showed UPV had the most significant effect on the UCS. We evaluated the criteria of the models (R2, MAE, RMSE), which showed high accuracy for estimating the UCS using these methods. Among the models, the Step-wise regression model showed the best results for forecasting the UCS. Comparing the results of the two models, the Step-wise regression model, with R2 = 0.99, MAE = 2.71, and RMSE = 3.60, showed the best performance for estimating the UCS of Charnockite rocks.

## Data Availability

The datasets used and/or analysed during the current study available from the corresponding author on reasonable request.
